# Loop-mediated isothermal amplification of *Neisseria gonorrhoeae* porA pseudogene: a rapid and reliable method to detect gonorrhea

**DOI:** 10.1186/s13568-017-0349-6

**Published:** 2017-02-23

**Authors:** Mei-Ling Liu, Yong Xia, Xing-Zhong Wu, Jian-Qiong Huang, Xu-Guang Guo

**Affiliations:** 1 Department of Clinical Laboratory Medicine, The Third Affiliated Hospital of Guangzhou Medical University, Guangzhou, 510150 China; 2 Department of Clinical Laboratory Medicine, Guangdong Center for Skin Disease & STI Control Guangzhou, Guangzhou, 510091 China; 3 KingMed School of Laboratory Medicine, Guangzhou Medical University, Guangzhou, 510150 China

**Keywords:** Gonorrhea, LAMP, *Neisseria gonorrhoeae* detection, porA gene

## Abstract

**Background and objective:**

Gonorrhea is a sexually transmitted disease caused by the bacterium *Neisseria gonorrhoeae*. Rapid detection is crucial for effective prevention and treatment. This study developed and tested a low-cost effective method for detecting *N. gonorrhoeae*, especially in developing countries.

**Methods:**

DNA from a *N. gonorrhoeae* standard strain, as well as from 26 genital secretion samples of gonorrhea patients, were isolated and used for loop-mediated isothermal amplification (LAMP) assay, which was conducted using either an automatic real-time PCR analyzer or a water bath. The amplified porA pseudogene sequence was compared with the NCBI database and the LAMP results were compared with that of the traditional culture method for its sensitivity and specificity.

**Results:**

LAMP was able to detect *Neisseria* DNA at a concentration as low as 1 pg/µL (1 × 10^3^ CFU/mL cells). The LAMP assay results obtained using an automatic real-time PCR analyzer was similar to that of the water bath. Relative to traditional culture, the sensitivity and specificity of the LAMP assay were 94.7 and 85.7%, respectively.

**Conclusion:**

LAMP was sensitive and reliable for detecting the porA gene of *N. gonorrhoeae*. It could be used as a rapid, low cost, and effective method for detecting *N. gonorrhoeae*.

## Introduction

Gonorrhea is a sexually transmitted disease caused by the bacterium *Neisseria gonorrhoeae*, a gram negative, obligate human pathogen. There are about 106 million incidences of gonorrheal infection each year worldwide (Organization 2012), and infection rates are about 10–20 times higher in developing countries than in developed countries (Van Der Pol et al. 2013).

Gonorrhea has been traditionally diagnosed by gram stain and culture, but testing methods based on polymerase chain reactions (PCRs) are becoming more common (Ng and Martin 2005; Barry and Klausner 2009; Deguchi et al. 2010). Traditional culture requires a specific growth media and is time-consuming, while PCR methods require specialized equipment that may not be readily available, especially in the hospitals of many developing countries.

Loop-mediated isothermal amplification (LAMP), a modified PCR technique, was first proposed in the year 2000 (Notomi et al. 2000). It uses Bst DNA polymerase, and 3-to-4 pairs of primers that are specific for 6 unique sequences of a target DNA template. The sensitivity and specificity of LAMP is similar to that of regular PCR, but LAMP requires only a water bath for the (Sirichaisinthop et al. 2011). Amplification of 1 × 10^10^ copies of original DNA template can be accomplished in a 65 °C water bath in 15–60 min. The product can be detected easily using various methods (Zhao et al. 2011; Li et al. 2014).

The success of PCR relies heavily on the target gene and its corresponding primers. Several target genes have been used for PCR involving *N. gonorrhoeae*, including 16S rRNA, cystine DNA methyltransferase, the cryptic plasmid (ccpB gene), opa, and porA pseudogene (Hjelmevoll et al. 2006). The porA pseudogene is conserved and highly specific to various strains of *Neisseria gonorrhoeae* (Whiley et al. 2006a, b). While the sensitivity of detection can be low because there is only a single copy in the genome (Mangold et al. 2007; Maze et al. 2011), this problem can be overcome by increasing the number of PCR cycles.

Due to its relative simplicity and low cost, LAMP has become a useful and popular method of diagnosis for many infectious diseases, especially in many developing countries (Macarthur 2009; Hopkins et al. 2013; Oriero et al. 2015). The aim of the present study is to test whether LAMP can be used as a possible method for rapid detection of *N. gonorrhoeae* using the porA pseudogene. Our report shows that LAMP can be used as a simple, cost-effective, and reliable method for gonorrhea diagnosis, especially in developing countries.

## Methods

The study was approved by the institutional ethics committee of Third Affiliated Hospital of Guangzhou Medical University. All patients provided written consent prior to sample collection.

### Clinical sample collection and other Bacterial strains

Samples were collected between March 2015 and 2016 from two hospitals in Guangzhou, southern China, namely, Third Hospital of Guangzhou Medical University, and Guangdong Institute of Dermatology.

Two genital secretion samples were collected from each patient using sterile swabs. One sample was used for traditional culture and the other for DNA isolation. Thayer-Martin (TM) agar plates (Beiruite Bio-technology, Zhengzhou, China) were used for traditional culture. Bacteria were grown on TM plates for 48 h at 36 °C in a growth chamber containing 5% CO_2_. The colony morphology and bacterial cells were examined using gram staining and microscopy (Ng and Martin 2005). Bacterial DNA was isolated, using a DNA isolation kit (Guangzhou Deaou Biotechnology) in accordance with the manufacture’s instructions. Isolated DNA was stored in Eppendorf tubes at −20 °C. Other bacterial strains used in this study are listed in Table 1.

**Table 1:** Bacterial strains used in this study

*Neisseria gonorrhoeae* ATCC49926
*Pseudomonas aeruginosa*
*Candida albicans*
*Candida glabrata*
*Enterobacter cloacae*
*Staphylococcus epidermidis*
*Acinetobacter baumannii*
*Viridans streptococci*
*Staphyloccocus aureus*
*Klebsiella pneumoniae*
*Streptococcus pneumoniae*
*Mycobacterium tuberculosis*
*Candida guilliermondii*
*Candida parapsilosis*
*Neisseria cinerea*
*Escherichia coli*
*Proteus mirabilis*
*Proteus penneri*
*Enterococcus faecalis*
*Enterococcus faecium*
*Candida lipolytica*
*Candida krusei*
*Candida norvegensis*

The source of all bacterial strains was: Third Affiliated Hospital of Guangzhou Medical University

### LAMP and color reaction

The sequence of the *N. gonorrhoeae* porA gene was obtained from GenBank. Primers (Table 2) were designed using Primer Explorer version 4 online software (http://primerexplorer.jp/e). The primers were synthesized by Invitrogen.

**Table 2:** Primers for the *Neisseria gonorrhoeae* porA gene

	Sequence (5′–3′)
F3	CCATTGATCCTTGGGACAG
B3	CAGACCGGCATAATACACAT
FIP(F1c + F2)	GGGAATCGTAACGCACGGAAATAATGTGGCTTCGCAATTG
BIP(B1c + B2)	AGCGGCAGCATTCAATTTGTTCCTGATTACTTTCCAGCGTGA
FLP	ATACCGTCGTGGCGTTTG
BLP	CGCCTATACGCCTGCTAC

The LAMP mix without DNA templates (Table 3) was first prepared on ice. Two microliters of DNA template was added to 23 µL of LAMP mix, to a total volume of 25 µL. One set of the final mix was amplified using an automatic real-time PCR analyzer (Cobas Z 480, Roche Molecular Diagnostics). The reaction condition was 45–60 min at 63 °C.

**Table 3:** Composition of the LAMP reaction mix

	Components	Volume
Reaction mix	20 mM Tris–HCl (pH 8.8)	12.5 µL
	10 mM KCl	
	8 mM MgSO_4_	
	10 mM (NH4)_2_SO_4_	
	0.1% Tween-20	
	1 mM Betaine	
	1.6 mM dNTP	
Primer mix	FIP (1.6 µM) and BIP (1.6 µM)	1 µL
	F3 (0.2 µM) and B3 (0.2 µM)	
	FLF (0.8 μM) and FLB (0.8 μM)	
Nuclease-free water		8 µL
Bst DNA polymerase		1 µL
SYTO-9		0.5 µL
DNA template		2 µL
Total volume		25 µL

Reaction condition: 63 °C, 45–60 min

Another set of the final mix was amplified using a water bath, and the fluorescence signal was detected directly by the naked eye. The reaction conditions were: 63 °C-water bath for 60 min, then 80 °C-water bath for 2 min to terminate the reaction. One microliter of SYTO-9 (Guangzhou Deaou Biotechnology) was added to the tubes. The reaction was considered positive if green florescence was observed.

### LAMP sensitivity test

DNA isolated from the *Neisseria gonorrhoeae* standard strain American Type Culture Collection (ATCC) 49926 was used to test the sensitivity of LAMP towards the porA gene. The DNA concentration of ATCC 49926 was measured and adjusted to 10 ng/µL using a spectrophotometer (Thermo NanoDrop 2000). The DNA was then diluted serially using sterile double-distilled water (ddH_2_O) to the following concentrations: 1 ng/µL; 100, 10, and 1 pg/µL; and 100 and 10 fg/µL. These concentrations correspond to the bacterial concentrations 1 × 10^6^, 1 × 10^5^, 1 × 10^4^, 1 × 10^3^, 1 × 10^2^, and 1 × 10^1^ CFU/mL, respectively.

The LAMP experiment was conducted as described above. Sterile ddH_2_O was used as the negative control and the 10 ng/µL DNA as a positive control. The lowest detectable concentration was determined. The LAMP experiment was then repeated using 20 different DNA templates isolated from the same *N. gonorrhoeae* standard strain (ATCC 49926), but each from a different colony, to further test for sensitivity and replicability (as described above). DNA isolated from 23 bacterial strains (Table 1) was also used to test the specificity of LAMP.

### LAMP test of clinical samples

Isolated DNA samples from 26 patients were used for porA-LAMP experiments, which were conducted using the automatic real-time PCR analyzer as well as a water bath. The results were then compared with that of the traditional culture method.

## Results

### Identification of *N. gonorrhoeae* by the traditional method

Samples of genital secretions were tested by the traditional method. The TM agar plate cultures showed that 19 samples (73.1%) tested positive for *Neisseria gonorrhoeae* and 7 were negative. After a 48-h culture, *N. gonorrhoeae* colonies were grayish-white-to-colorless mucoid. Gram staining showed that the bacterial cells were gram negative.

### Reliability and sensitivity of LAMP for amplification of *N. gonorrhoeae* porA pseudogene

**Fig. 1:**
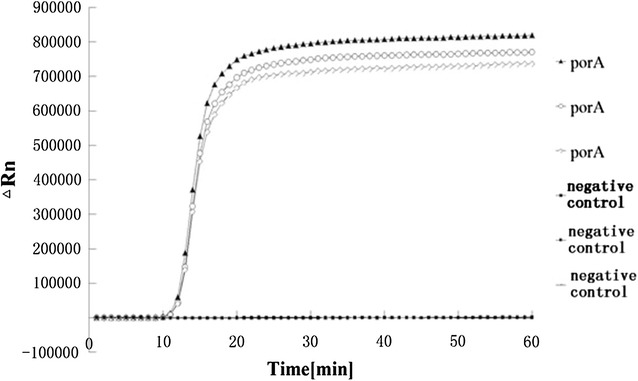
LAMP detection of the ATCC 49926 porA gene using an automatic PCR cycler

**Fig. 2:**
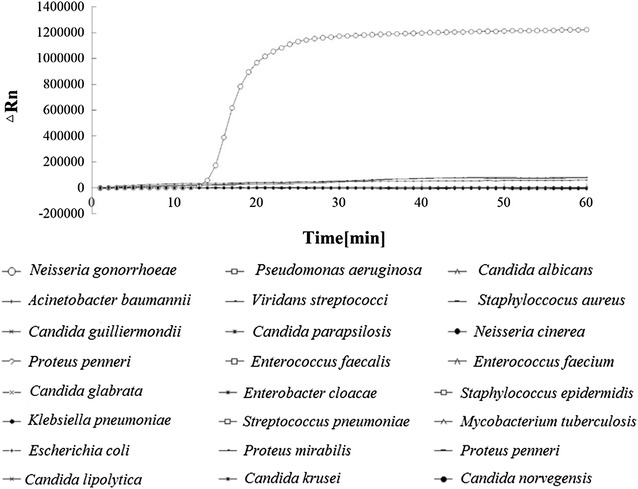
Specificity of LAMP for porA detection. The LAMP product (fluorescence) was detected from DNA of the *Neisseria gonorrhoeae* standard strain, but not in any of the 23 non-*Neisseria* bacteria

Using DNA isolated from the *N. gonorrhoeae* standard strain ATCC 49926, we first tested the reliability and sensitivity of LAMP for porA amplification (Fig. 1). Amplification was detected 10 min after the reaction and reached its peak at around 20 min. No amplification was detected in the negative control (ddH_2_O). This indicates that LAMP is reliable for porA amplification. In addition, we found that the porA primers were specific to *N. gonorrhoeae*. No fluorescence (LAMP product) was detected when DNA from the 23 bacterial species (Table 1) was used as a template (Table 1; Fig. 2).

**Fig. 3:**
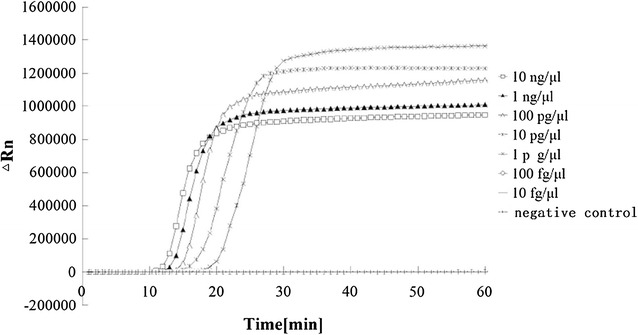
Sensitivity test for LAMP using different concentrations of DNA templates from *Neisseria gonorrhoeae* ATCC 49926

To test the sensitivity of the LAMP reaction, 10-fold serial dilutions of the DNA template (i.e., from 1 ng/µL to 10 fg/µL) were used for the LAMP experiment (Fig. 3). Amplification was detected 18 min after the reaction, when the initial DNA template was as low as 1 pg/µL.

**Fig. 4:**
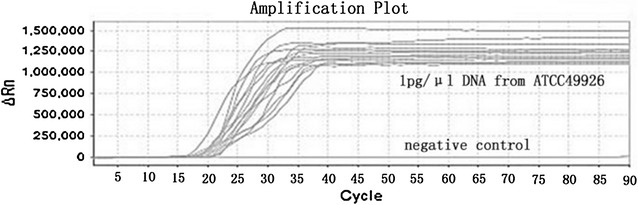
LAMP assay using 20 different DNA templates isolated from the same *Neisseria gonorrhoeae* standard strain (ATCC 49926), but from different colonies, at a concentration of 1 pg/µL

To further confirm the reliability of LAMP, the experiment was repeated using 20 different DNA templates isolated from the same *N. gonorrhoeae* standard strain (ATCC 49926), but each from a different colony, at a concentration of 1 pg/µL. Amplification products were detected from all 20 samples (Fig. 4), further indicating that LAMP is very sensitive in detecting the porA gene. No product was detected when the DNA template was replaced with ddH_2_O.

### LAMP analysis of clinical samples

**Fig. 5:**
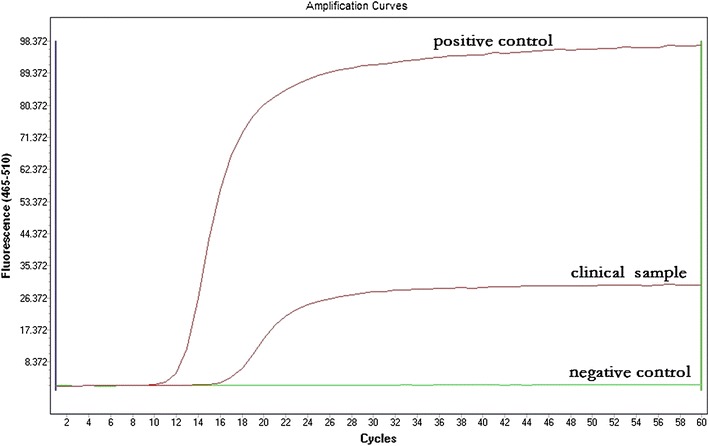
A representative example of LAMP using the clinical sample and the positive control (DNA from ATCC 49926)

**Fig. 6:**
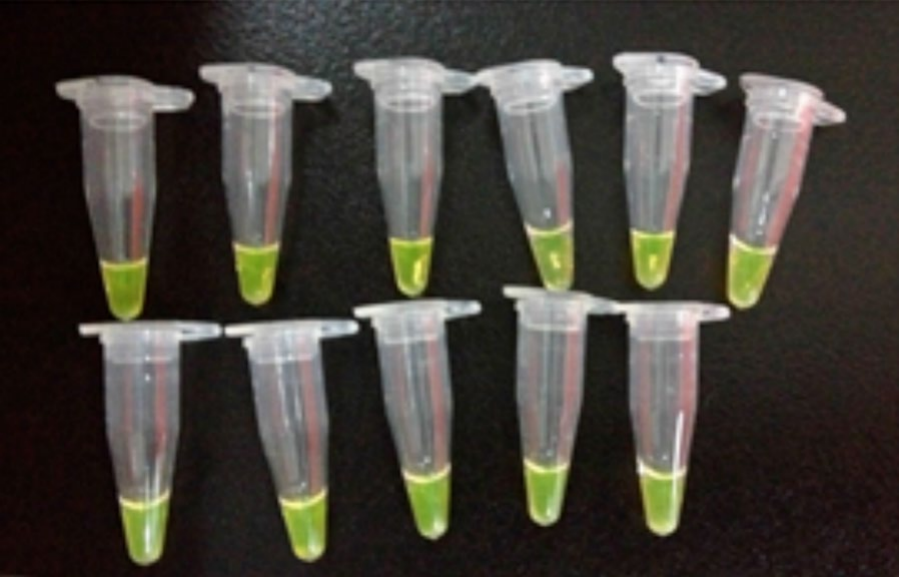
LAMP assay using a water bath and direct observation of the LAMP products via fluorescence dye

LAMP was used to detect porA genes from the 26 clinical samples (Fig. 5). Nineteen (73.1%) of the clinical samples tested positive for the porA gene and 7 samples were negative. One of the 19 Neisseria-positive by traditional culture sample was LAMP-negative (false negative), and one of 7 Neisseria-negative by traditional culture sample was LAMP-positive (false positive). Compared to traditional culture, the sensitivity and specificity of the LAMP assay were 94.7 and 85.7%, respectively. The initial detection time for the positive control was shorter (16 min) than that of the clinical sample (19 min; Fig. 5), possibly because of the higher concentration of the DNA template of the positive control. A similar result was obtained using a water bath for LAMP (Fig. 6).

## Discussion

Improvements of the LAMP technique have been made constantly since its inception in 2000 (Notomi et al. 2000). The goal of this study was to develop and test a low-cost, effective method for detecting *N. gonorrhoeae*, especially a method that could be used in developing countries. We showed that LAMP is both reliable and sensitive for detecting the *Neisseria* porA gene, either by using the *N. gonorrhoeae* standard strain (ATCC 49926) or clinical DNA samples isolated from genital secretions of gonorrhea patients. This is consistent with a previous study, which detected the glutamine synthetase gene of *N. gonorrhoeae* from urine samples (Edwards et al. 2014). That study also showed that the LAMP assay was able to tolerate a urea concentration up to 1.8 M, while conventional PCR tolerates only a urea concentration no more than 100 mM. Another study showed that better detection results were obtained using an endocervical swab than urine samples (Gaydos et al. 2003).

The *N. gonorrhoeae* genome has been sequenced and studied extensively (Dempsey et al. 1991; Dempsey and Cannon 1994; Chung et al. 2008). Having the genome greatly facilitated diagnosis of gonorrhea, especially through molecular techniques such as PCR. Many commercial kits, such as the Aptima Combo 2 assay (Gaydos et al. 2003) and the COBAS AMPLICOR *N. gonorrhoeae* PCR kit (Luijt et al. 2005), have been developed with satisfactory detection rates. All these techniques require selection of the appropriate target genes (Hjelmevoll et al. 2006). In the current study, we chose the highly conserved porA pseudogene as the LAMP target gene (Whiley et al. 2006a). PorA is the only outer membrane porin gene found in the *N. gonorrhoeae* genome and contains a very low-level of genetic polymorphism (Unemo et al. 2005). Because of its high efficiency of amplification, porA can be detected quickly, although *N. gonorrhoeae* has only a single copy of porA in the genome (Hjelmevoll et al. 2006; Whiley et al. 2006b). Consistently, we found that the porA gene was detected 10 min after the reaction and reached its peak at 20 min.

Traditional culture is still used and is considered the gold standard for identifying *N. gonorrhoeae* infection. However, the sensitivity is low and the method is time-consuming (Caliendo et al. 2013). In addition, it is not suitable for patients who are under antibiotic treatment. In the present study, we found that the detection rates of the LAMP assay and traditional culture were similar (~70%). Using traditional culture as a standard, one false positive and one false negative were found among the 26 clinical samples (3.8%) by LAMP assay; and the sensitivity and specificity of the LAMP assay reached 94.7 and 85.7%, respectively. The high sensitivity of LAMP is more impressive when testing with the *N. gonorrhoeae* standard strain, detecting as low as 1 pg/µL of the DNA concentration, the equivalent of 1 × 10^3^ CFU/mL. This sensitivity was further confirmed with 20 parallel samples of the standard strain (Fig. 4). Compared to the standard strain, the initial detection time of the clinical samples was slower (19 min compared with 16 min). This is likely caused by differences in concentration and purity, as the DNA of the standard strain was isolated directly from pure bacterial culture, and the clinical samples contained a lower bacterial load and the isolated DNA was not as pure as that of the standard strain.

The LAMP method requires much less time than traditional culture, and is also more cost-effective. In this study, we showed that the LAMP assay was accomplished in simple water baths and the reaction products could be visualized simply with the naked eye (Fig. 6). In brief, our study suggests that LAMP could be used as a simple, cost-effective, and reliable method for the diagnosis of gonorrhea, especially in developing countries. One limitation of the study is that the clinical sample size is relatively small. A larger and more diverse samples could better determine the sensitivity and reliability of the method.
